# A Polyamine-Deficient Diet Prevents Oxaliplatin-Induced Acute Cold and Mechanical Hypersensitivity in Rats

**DOI:** 10.1371/journal.pone.0077828

**Published:** 2013-10-30

**Authors:** Jérémy Ferrier, Mathilde Bayet-Robert, Bruno Pereira, Laurence Daulhac, Alain Eschalier, Denis Pezet, Jacques-Philippe Moulinoux, David Balayssac

**Affiliations:** 1 Clermont Université, Université d’Auvergne, Pharmacologie Fondamentale et Clinique de la Douleur, Clermont-Ferrand, France; 2 INSERM, U1107 NEURO-DOL, Clermont-Ferrand, France; 3 CHU Clermont-Ferrand, Clermont-Ferrand, France; 4 Groupe de Recherche en Thérapeutique Anticancéreuse, EA 3891, Université de Rennes 1, Rennes, France; University of Arizona, United States of America

## Abstract

**Background:**

Oxaliplatin is an anticancer drug used for the treatment of advanced colorectal cancer, but it can also cause painful peripheral neuropathies. The pathophysiology of these neuropathies has not been yet fully elucidated, but may involve spinal N-methyl-D-aspartate (NMDA) receptors, particularly the NR2B subunit. As polyamines are positive modulators of NMDA-NR2B receptors and mainly originate from dietary intake, the modulation of polyamines intake could represent an interesting way to prevent/modulate neuropathic pain symptoms by opposing glutamate neurotransmission.

**Methods:**

The effect of a polyamine deficient diet was investigated in an animal model of oxaliplatin-induced acute pain hypersensitivity using behavioral tests (mechanical and cold hypersensitivity). The involvement of spinal glutamate neurotransmission was monitored by using a proton nuclear magnetic resonance spectroscopy based metabolomic approach and by assessing the expression and phosphorylation of the NR2B subunit of the NMDA receptor.

**Results:**

A 7-day polyamine deficient diet totally prevented oxaliplatin-induced acute cold hypersensitivity and mechanical allodynia. Oxaliplatin-induced pain hypersensitivity was not associated with an increase in NR2B subunit expression or phosphorylation, but with an increase of glutamate level in the spinal dorsal horn which was completely prevented by a polyamine deficient diet. As a validation that the oxaliplatin-induced hypersensitivity could be due to an increased activity of the spinal glutamate system, an intrathecal administration of the specific NR2B antagonist, ifenprodil, totally reversed oxaliplatin-induced mechanical and cold hypersensitivity.

**Conclusion:**

A polyamine deficient diet could represent a promising and valuable nutritional therapy to prevent oxaliplatin-induced acute pain hypersensitivity.

## Introduction

Oxaliplatin is a third-generation platinum derivative that has become the standard treatment for advanced colorectal cancers [1]. However, oxaliplatin also has a unique pattern of neurotoxicity. In more than 90% of patients, oxaliplatin induces an acute and transient thermal hypersensitivity, with a rapid onset of distal paresthesias and cold-triggered dysesthesias [2], [3]. Thereafter, with the repetition of chemotherapy cycles, 50–80% of patients experience a severe cumulative peripheral neuropathy that is poorly reversible [4], [5]. As a consequence, oxaliplatin-induced neurotoxicity is strongly incapacitating for patients and affects their quality of life [5], [6]. Moreover, the lack of neuroprotective strategies to prevent these peripheral neuropathies often compels oncologists to reduce or discontinue the chemotherapy cycles, thus decreasing the chances of survival [7].

The exact cause of this neurotoxicity is yet to be conclusively identified. Mihara *et al.* (2011) recently showed that repeated administrations of oxaliplatin induced N-methyl-D-aspartate receptor subtype 2B (NR2B) up-regulation in the rat spinal cord [8], which is known to be involved in the development and maintenance of chronic pain resulting from peripheral nerve injury [9]–[11]. However, the clinical use of selective NR2B antagonists remains limited by severe adverse effects, despite their analgesic efficacy in neuropathic pain [12]. It has recently been shown that a polyamine-deficient diet (PDD) relieved pain hypersensitivity in animal models of chronic mononeuropathy and monoarthritis and decreased tyrosine phosphorylation of the NR2B subunit in the rat spinal cord after intraplantar carrageenan injection [13]. Polyamines are biogenic polycations ubiquitously distributed in eukaryotic cells. The main source of polyamines is exogenous, through dietary intake and absorption of the gut flora metabolites [14]. An endogenous source is also provided by the decarboxylation of ornithine *via* ornithine decarboxylase (ODC) and by interconversion pathways [15]. Polyamines (including spermine, spermidine and putrescine) have been extensively studied as positive modulators of NR2B-containing NMDA receptor activity [16], thus facilitating pain sensitization [13].

The aim of the present study was to validate the concept that a PDD could be a beneficial strategy to reduce oxaliplatin-induced acute pain hypersensitivity by inhibiting glutamate neurotransmission in the spinal cord. First, the preventive effect of a PDD on oxaliplatin-induced acute pain hypersensitivity was investigated in an animal model of oxaliplatin-induced acute hypersensitivity using behavioral tests. Secondly, the involvement of spinal glutamate neurotransmission in this animal model was monitored by using a ^1^H-NMR-based metabolomic approach and assessing the expression and phosphorylation of the NR2B subunit. Thereafter, the impact of PDD on oxaliplatin-induced increase of spinal glutamate levels was explored. Finally, the implication of the NR2B subunit was confirmed by evaluating the antihyperalgesic effect of ifenprodil, a NR2B-selective antagonist, on oxaliplatin-induced acute cold and mechanical pain hypersensitivity.

## Materials and Methods

### Ethics Statement

All experiments involving animals were performed in compliance with EU legislation (directive 86/609/EEC of 24 November 1986), and all procedures were approved by the local ethical review board for animal experiments (CEMEA - *Comité d’Ethique en Matière d’Expérimentation Animale Auvergne*) under agreement numbers CE14-11, CE26-10 and CE16-10.

### Animals

The experiments used adult male Sprague-Dawley rats (Charles River, France) weighing 150 to 175 g at the beginning of the study. The rats were housed in a controlled environment (22°C ±2°C; 50% relative humidity; 12/12 light-dark cycle) in groups of 4 rats per cage. To minimize stress during the behavioral tests, the rats were given an acclimatization period of 7 days to habituate them to the handling and testing procedures.

### Diet

The synthetic diet was prepared with products from Sigma Chemical Co. (Sigma, France) according to the formulation of Kergozien *et al.* [17]. Two different synthetic diets were used in this study: (1) a normal diet providing an equivalent amount of polyamines to normal rat chow (54 mg/kg putrescine, 37 mg/kg cadaverine, 27 mg/kg spermidine and 7 mg/kg spermine) and fulfilling all rodent nutritional needs [17]; (2) a polyamine-deficient diet (PDD) equivalent to normal rodent chow but containing less than 10 µg/kg of polyamines. Dietary polyamine level was determined by atmospheric-pressure chemical ionization as previously described [18].

On reception at the animal housing facility, *i.e*. 7 days before the beginning of the experiments, the rats were assigned to a specific diet (normal diet or PDD) and left to become habituated to the experimental environment. Food and water were provided *ad libitum.*

### Animal Model of Oxaliplatin-induced Acute Pain Hypersensitivity

Acute neurotoxicity was induced in rats by a single intraperitoneal (i.p.) oxaliplatin injection (6 mg/kg), as described by Ling *et al.* [19]. Oxaliplatin (Debiopharm, Switzerland) was dissolved in a 5% glucose solution (Freeflex®, Fresenius Kabi, France) at a final concentration of 2 mg/ml. Control rats received an equal i.p. injection of vehicle.

### Behavioral Testing Procedures of Hypersensitivity

Behavioral tests were chosen according to clinical symptoms (mechanical allodynia/hyperalgesia and cold hypersensitivity) observed in oxaliplatin-treated patients [20], [21]. The electronic von Frey test was used to assess mechanical allodynia/hyperalgesia and the thermal place preference test was used to measure cold avoidance behavior.

#### Mechanical allodynia/hyperalgesia

The electronic von Frey test (Bioseb®, France) consists of a hand-held force transducer fitted with a plastic tip. Each rat was placed in a suspended plastic cage (17×11×13 cm) with a wire grid floor 15 min before the beginning of the test. The plastic tip was applied perpendicularly to the medial plantar surface of the hindpaw from below the wire grid floor. The force applied was gradually increased until withdrawal of the hindpaw. Mechanical pressure thresholds (g) were automatically registered by the electronic device and used as a pain parameter.

#### Cold hypersensitivity

The thermal place preference test (Bioseb®, France) was used to assess a cold avoidance behavior. This test consists of two contiguous metal plates surrounded by a plastic enclosure. The first plate was kept at neutral temperature (25°C) whereas the second plate was kept at cold temperature (12°C, determined by preliminary experiments). The test was performed in the morning, and in darkness. Each session lasted 3 minutes, during which the animals were left free to explore both plates. The time spent on the cold plate during the entire session was recorded using an infrared camera connected to a computer in order to determine cold avoidance behavior. To improve the explorative behavior of the rats used for this test, the light-dark cycle was inverted immediately after the animal arrived at the animal housing facility. A few days before the beginning of the experiment, the rats were repeatedly placed on the apparatus with both plates held at room temperature (25°C) during 3 minutes. In these conditions, rats spent an equal amount of time on each plate, thus showing no place preference. To avoid learning or any place preference unrelated to cold, the temperature of the plates were inverted between two consecutive sessions.

### Immunoblotting

Dorsal spinal cord samples were homogenized in 400 µL of ice-cold lysis buffer (50 mM HEPES, pH 7.5, 150 mM NaCl, 10 mM EDTA, 10 mM Na_4_P_2_O_7_, 2 mM orthovanadate, 100 mM NaF, 1% Triton X-100, 0.5 mM PMSF, 20 µM leupeptin, and 100 IU/mL aprotinin; Sigma, St-Quentin-Fallavier, France), as described in [22]. After 1–2 min sonication at 4°C, samples were centrifuged (16,000 *g*, 15 min, 4°C), and the pellets were discarded. Protein concentration was determined using a bicinchoninic acid protein kit assay (BC Assay UP40840A®, Interchim, France). Protein content was then separated in 7.5% SDS-PAGE and transferred to a nitrocellulose membrane (Millipore, France). Membranes were blotted overnight at 4°C with the antibodies indicated according to the manufacturer’s recommendations. Tested antibodies were total NR2B (1∶500, ref: 06–600, Upstate Biotechnology, Millipore, France), phospho-Tyr1472 (1∶500, ref: AB5403, Millipore, France), phospho-Tyr1336 (1∶500, ref: AB9690, Millipore, France) and phospho-Ser1303 (1∶500, ref: 07–398, Millipore, France). The next day, the membranes were blotted with horseradish peroxidase-conjugated secondary antibodies (1∶50,000, goat anti-rabbit, Pierce, France). Immunoreactivity was detected using a chemiluminescence kit (Immun-star™ WesternC™ Kit, Bio-rad®, France). Representative western blots were scanned using a ChemiDoc™ XRS system (Bio-rad®, France). The density of each band was quantified using Image Lab™ software (Biorad®, France) and normalized against the corresponding beta-actin signal used as a loading control (1∶5000, A5441, Sigma-Aldrich, France). The resulting ratios were expressed as percentage of control (vehicle).

### Proton NMR Spectroscopy-based Metabolomic Analysis

#### Sample preparation

Spinal dorsal horn from 2 rats receiving the same regimen were pooled to reach a minimum of 80 mg of tissue (n = 8 to 10 pooled by two, i.e. n = 4 to 5 for each group). Aqueous metabolite extraction was performed as described by Angenstein *et al.* [23], with minor changes. Briefly, tissue samples were homogenized in a 6% perchloric acid solution (5 ml/g of tissue) and centrifuged (12,000 *g*, 10 min, 4°C). The supernatant was adjusted to pH 11 using a 10% potassium hydroxide solution. Potassium perchlorate precipitate was then removed by centrifugation (4,000 *g*, 10 min, 4°C). Samples were lyophilized overnight and stored at −80°C until NMR spectroscopy analysis. Dried samples were reconstituted in 600 µl D_2_O containing 1 mM of 3-(trimethylsilyl)-2,2,3,3-tetradeuteropropionic acid (TSP, Sigma, France) used as internal standard for concentration and chemical shift.

#### Data acquisition and processing

Experiments were performed on a Bruker Avance 400MHz spectrometer (Bruker Biospin, Germany) equipped with a quad-nuclear probe. Spectral acquisitions were automated using ICON-NMR software (Bruker BioSpin GmbH, Germany). 1D ^1^H-NMR spectra were obtained using a Nuclear Overhauser Enhancement SpectroscopY sequence with low-power water signal PResaturation (NOESYPR), a 2.5-s presaturation delay, and a 1-s relaxation delay. Spectral width was 12 ppm with 16K complex datapoints and 128 repetitions. After Fourier transformation, low-order phase correction and spline baseline correction were applied in a standardized way. 1D ^1^H-NMR spectra were processed using TopSpin 3.1 (Bruker BioSpin GmbH, Germany).

#### Metabolite identification and quantification

**Figure 1:**
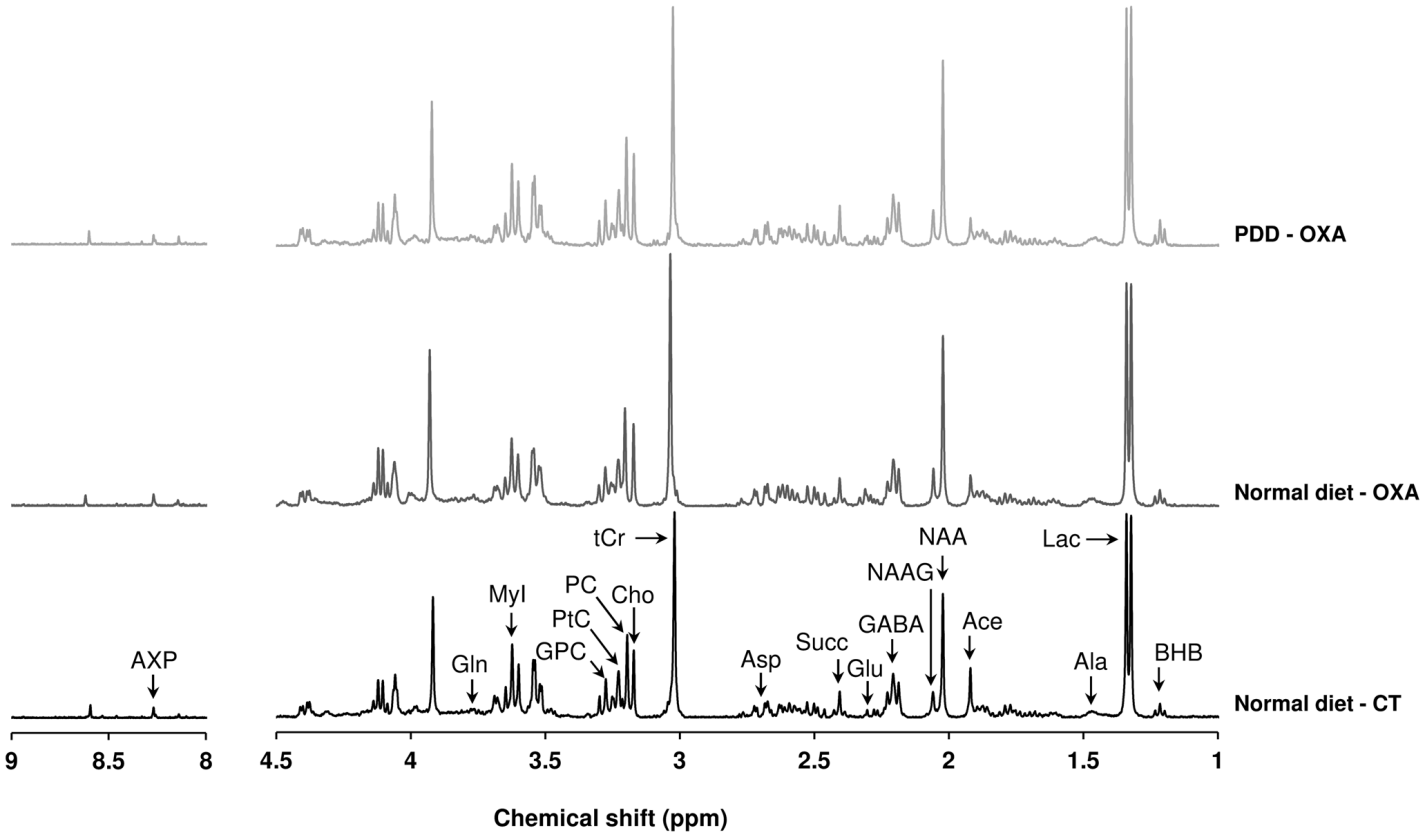
Representative 1D ^1^H-NMR spectra of perchloric acid extracts from rat spinal dorsal horn. A representative NMR spectrum is displayed for samples extracted from control rats fed with a normal diet (normal diet - CT) (bottom panel, black), oxaliplatin-treated rats fed with a normal diet (normal diet - OXA) (middle panel, dark grey) and oxaliplatin-treated rats fed with a PDD (PDD - OXA) (upper panel, light grey). Chemical shifts are expressed in parts per million (ppm). Eighteen metabolites were identified and quantified (Ace, acetate; Ala, alanine; Asp, aspartate; AXP, adenosine phosphates; BHB, β-hydroxybutyrate; Cho, choline; GABA, γ-aminobutyric acid; Gln, glutamine; Glu, glutamate; GPC, glycerophosphocholine; Lac, lactate; MyI, myo-Inositol; NAA, N-acetylaspartate; NAAG, N-acetylaspartylglutamate; PC, phosphocholine; PtC, phosphatidylcholine; Succ, succinate; tCr, total creatine).

Representative high-resolution 1D ^1^H-NMR spectra of the metabolite extract from control and oxaliplatin-treated rats fed with a normal diet and oxaliplatin-treated rats fed with a PDD are presented in Figure 1. Four to 5 ^1^H-NMR spectra were recorded for each treatment condition. Eighteen metabolites were identified and quantified in each ^1^H-NMR spectrum. Metabolite concentrations were measured by peak integration and compared to the integral of the reference compound TSP 1 mM in each spectrum. Metabolite concentration, expressed as a percentage of control fed with a normal diet, was calculated as follows: [mean metabolite concentration in a treated group/mean metabolite concentration in the control group fed with a normal diet]×100.

### Experimental Design

For *in vivo* studies, four different groups of diet and treatment were defined to determine the effect of a PDD on oxaliplatin-induced acute nociceptive disorders. They included control rats fed with a normal diet or PDD, and oxaliplatin-treated rats fed with a normal diet or PDD. The inhibition of NR2B-containing NMDA receptors was evaluated in oxaliplatin-treated rats fed with a normal diet with the specific inhibitor ifenprodil. Ifenprodil tartrate (Sigma, France) was dissolved in a saline solution (0.9% NaCl) just before administration. At 3 days post-oxaliplatin injection, neuropathic rats were randomly assigned to 4 groups (n = 8 per group) according to ifenprodil dose administered: 0 µg (NaCl 0.9%), 2.5 µg/rat, 5 µg/rat or 10 µg/rat. Ifenprodil was intrathecally administered under isoflurane anesthesia at a final volume of 10 µL/rat. Experiments were performed blind with randomization of treatments and according to the method of equal blocks to avoid any uncontrollable environmental changes.

For *ex vivo* analyses, rats were sacrificed by decapitation at day 3 (peak pain behavior) post-oxaliplatin injection (Ling *et al.*, 2007). Sacrifices were performed by a trained professional, without any anesthetic that could modify spinal metabolism. The spinal cords (L4–L6) were quickly removed, and the dorsal part of the spinal cord were isolated on an ice pack, immediately frozen in liquid nitrogen, and stored at −80°C until analysis. The total procedure lasted less than 5 min for each sample.

### Statistical Analysis

In behavioral tests, means and standard error of the mean (SEM) were calculated for quantitative variables. Normality of the distribution was checked with a Shapiro-Wilk test. To compare the time-course evolution of different parameters, a repeated-measure ANOVA (or a non-parametric Friedman test if necessary) was followed by a Tukey-Kramer test to compare differences between and within groups. If a significant interaction between time and group was observed, a one-way ANOVA was performed for all time points. These analyses were completed by random-effects models (REM), considered more robust to missing data [24]. The REM were able to take into account 1) fixed effects as treatment, time and interaction between time and group, and 2) random subject effects as random intercept and slope. Residual normality was checked for all models presented in this article. Results from immunoblotting were analyzed using a nonparametric Mann-Whitney-Wilcoxon test to compare differences between groups. Differences were considered statistically significant at p<0.05. Statistical analysis was performed using STATA® v.10 software (StataCorp, College Station, TX, USA).

In the metabolomic analysis, statistical comparisons between control and treated groups were performed using the nonparametric Mann-Whitney test. Differences were considered statistically significant at p<0.05. To identify the most discriminating metabolites of response to oxaliplatin, PDD or the combination of both, partial least squares discriminant analysis (PLS-DA) was applied to concentrations of the 18 identified metabolites using XLSTAT v2012.6.08 software (Addinsoft). Coefficients of PLS-DA loading scores were used to express the level of correlation between original variables and components of the PLS-DA model. Cross-validation of each model led to an evaluation of the R_2_Y and Q_2_ factors. R_2_Y provides an estimate of how well the model fits the Y data, and Q_2_ provides an estimate of how well the model predicts the Y data. Typically, a robust model will have R_2_Y>0.5 and Q_2_>0.4. In PLS-DA, the variable influence on the projection (VIP), a weighted sum of squares of the PLS weights, was calculated to indicate the importance of a metabolite in the model. A VIP value clearly above 1.2 was required to retain the discriminating power of a metabolite.

## Results

No rat displayed any loss of weight or altered clinical condition regardless of the treatment or diet received (data not shown). There was no significant difference in daily food intake between rats fed with a normal diet (6.46±0.33 g/day/100 g body weight) and rats fed with a PDD (6.32±0.32 g/day/100 g body weight) (p = 0.56). Before oxaliplatin injection, there was no statistical difference between mean thresholds of the different groups in behavioral tests.

### The PDD Prevents Oxaliplatin-induced Mechanical and Cold Hypersensitivity

**Figure 2:**
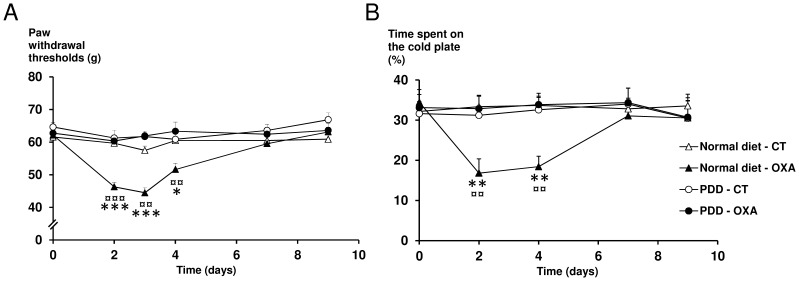
Effect of a PDD on oxaliplatin-induced mechanical and cold hypersensitivity. Rats were fed with a normal diet (triangles) or a PDD (circles) for 7 days before the beginning of the tests and through to the end of the experiment. At day 0, after measuring baseline mechanical pain thresholds, rats were injected with oxaliplatin (6 mg/kg, i.p., OXA, black symbols) or vehicle (CT, white symbols). (A) Mechanical pain hypersensitivity expressed as paw withdrawal threshold (gram). (B) Cold hypersensitivity (12°C *vs.* 25°C) expressed as time spent on the cold plate (%). Two-way repeated-measures ANOVA followed by a Tukey-Kramer *post hoc* test. *, p<0.05; **, p<0.01; ***, p<0.001 for normal diet - OXA *vs*. normal diet - CT; ^¤¤^, p<0.01; ^¤¤¤^, p<0.001 for normal diet - OXA *vs.* PDD - OXA. All data are expressed as means ± SEM (n = 6 to 12 per group).

The PDD for 7 days did not modify sensitive thresholds compared to control rats fed with a normal diet. While, with a normal diet, oxaliplatin treated rats showed a significant decrease in paw withdrawal thresholds from day 2 (−22%, p<0.001) to day 4 (−15%, p = 0.016), compared to control rats, with a maximum reduction at day 3 (−23%, p<0.001) (Figure 2-A), oxaliplatine-treated rats fed with a PDD exhibited no significant change in mechanical thresholds. Analysis of REM showed a significant interaction between treatments and diets (p = 0.002).

Regarding oxaliplatin-induced cold hypersensitivity, while oxaliplatin treated rats fed with a normal diet showed a significant decrease in the time spent on the cold plate from day 2 (−50%, p<0.008) to day 4 (−45%, p<0.002), compared to controls (Figure 2-B), oxaliplatin-treated rats fed with a PDD showed no difference compared to control rats regardless of the diet received (normal diet or PDD). Analysis of REM showed a significant interaction between treatments and diets (p = 0.006).

### The PDD Suppresses Oxaliplatin-induced Increase of Glutamate Level in Spinal Dorsal Horn

#### Oxaliplatin increases spinal glutamate concentration without modifying NR2B expression or phosphorylation

**Figure 3:**
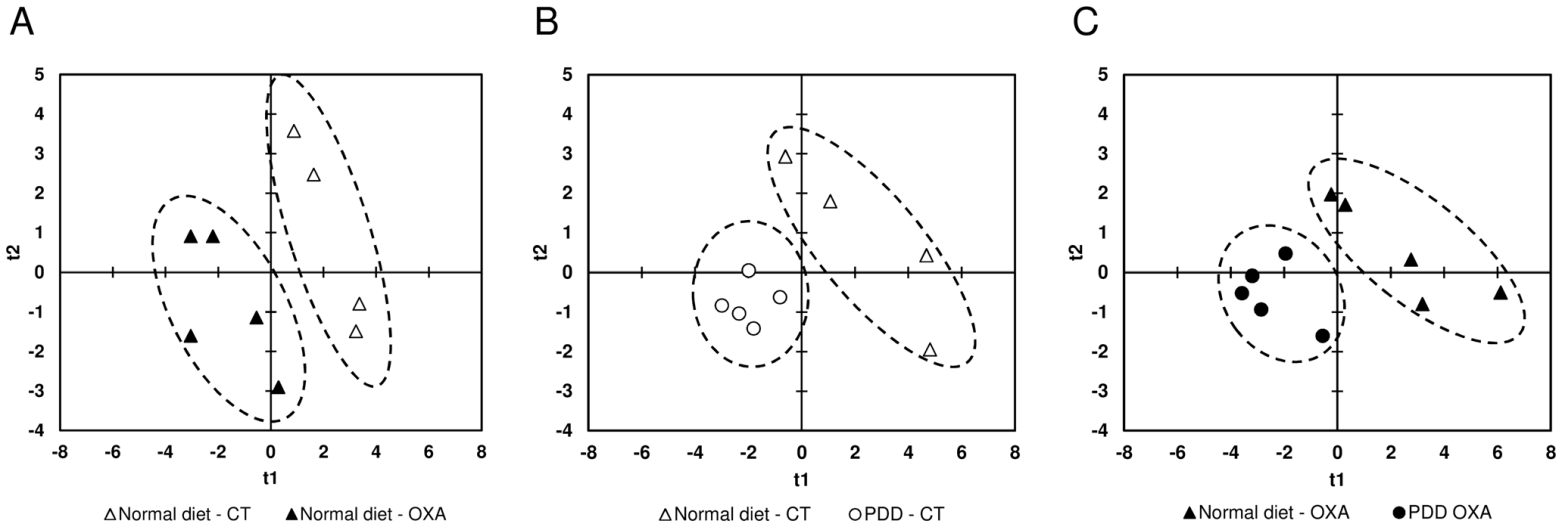
Score plots of PLS-DA analysis based on the 18 metabolites of the spinal dorsal horn quantified from ^1^H-NMR data. PLS-DA analysis revealed a clear separation of the metabolic phenotypes of spinal samples between control rats fed with a normal diet (normal diet - CT) and oxaliplatin-treated rats fed with a normal diet (normal diet - OXA) (A), control rats fed with a normal diet (normal diet – CT) and control rats fed with a PDD (PDD - CT) (B) and oxaliplatin-treated rats fed with a normal diet (normal diet - OXA) and oxaliplatin-treated rats fed with a PDD (PDD - OXA) (C) 3 days after oxaliplatin injection. Each point represents an individual sample. Ellipses have been drawn to encompass samples from the same treatment group (n = 4 to 5 per group).

Three days after oxaliplatin injection, there was a significant increase in glutamate (Glu, +85%, p = 0.0253) and a decrease in total creatine (tCr) (−21%, p = 0.0339) and adenosine phosphate (AXP) (−20%, p = 0.0143) concentrations in oxaliplatin treated rats compared to control rats fed with a normal diet (Table 1). PLS-Discriminant Analyses (PLS-DA) were performed on samples taking into account the treatment and diets received to identify relevant potential metabolites involved in oxaliplatin-induced metabolic changes. For animals fed with a normal diet, PLS-DA showed a clear separation between the samples of oxaliplatin treated and control animals (R_2_Y = 0.721, Q_2_ = 0.527, validated model, Figure 3-A), indicating that the metabolic fingerprints of these two groups are significantly distinct. The most discriminant metabolites with a VIP value above 1.2 were acetate (Ace), AXP, Glu, glycerophosphocholine (GPC), phosphatidylcholine (PtC) and tCr.

**Table 1:** Metabolites identification and quantification from 1D ^1^H-NMR spectra of rat spinal cord dorsal horn.

	Normal diet		Polyamine-deficient diet	
Metabolite	Control	Oxaliplatin	Control	Oxaliplatin
Acetate	0,324±0,063	0,206±0,063	0,407±0,168*	0,331±0,138*
Adenosine phosphates	0,210±0,015	0,168±0,023*	0,163±0,016*	0,142±0,021
Alanine	0,346±0,143	0,365±0,058	0,380±0,045	0,307±0,036*
Aspartate	0,729±0,262	1,011±0,351	0,652±0,116	0,651±0,116$
β-hydroxybutyrate	0,504±0,085	0,502±0,051	0,526±0,056	0,492±0,059
Choline	1,315±0,065	1,225±0,158	1,193±0,070	1,077±0,128
γ-aminobutyric acid	2,294±0,098	2,069±0,293	2,266±0,112	1,838±0,155*
Glutamate	0,385±0,240	0,711±0,242*	0,318±0,019	0,292±0,083$
Glutamine	0,577±0,196	0,578±0,071	0,488±0,050	0,483±0,032$
Glycerophosphocholine	0,345±0,055	0,285±0,014	0,267±0,016*	0,232±0,025* $
Lactate	6,629±0,481	5,718±0,881	5,606±0,278*	4,787±0,517*
*myo*-Inositol	3,011±0,220	2,629±0,332	2,572±0,229*	2,289±0,204
N-acetylaspartate	2,435±0,493	2,272±0,371	2,140±0,190	1,721±0,156* $
N-acetylaspartylglutamate	0,618±0,174	0,568±0,086	0,479±0,042	0,433±0,054$
Phosphatidylcholine	0,730±0,096	0,615±0,057	0,549±0,053*	0,499±0,035$
Phosphocholine	1,024±0,116	0,958±0,103	0,931±0,054	0,832±0,081$
Succinate	0,376±0,097	0,439±0,091	0,351±0,055	0,350±0,082
Total creatine	3,337±0,256	2,635±0,280*	2,681±0,199	2,517±0,197

The 18 identified metabolite concentrations of rats fed with a normal diet (control and oxaliplatin-treated) and rats fed with a PDD (control and oxaliplatin-treated), at day 3, are expressed in mmol/kg (means ± SEM, n = 4 to 5). Non-parametric Mann-Whitney-Wilcoxon test.

* p<0.05 *vs.* control rats fed with a normal diet;

$ p<0.05 *vs.* oxaliplatin-treated rats fed with a normal diet.

**Figure 4:**
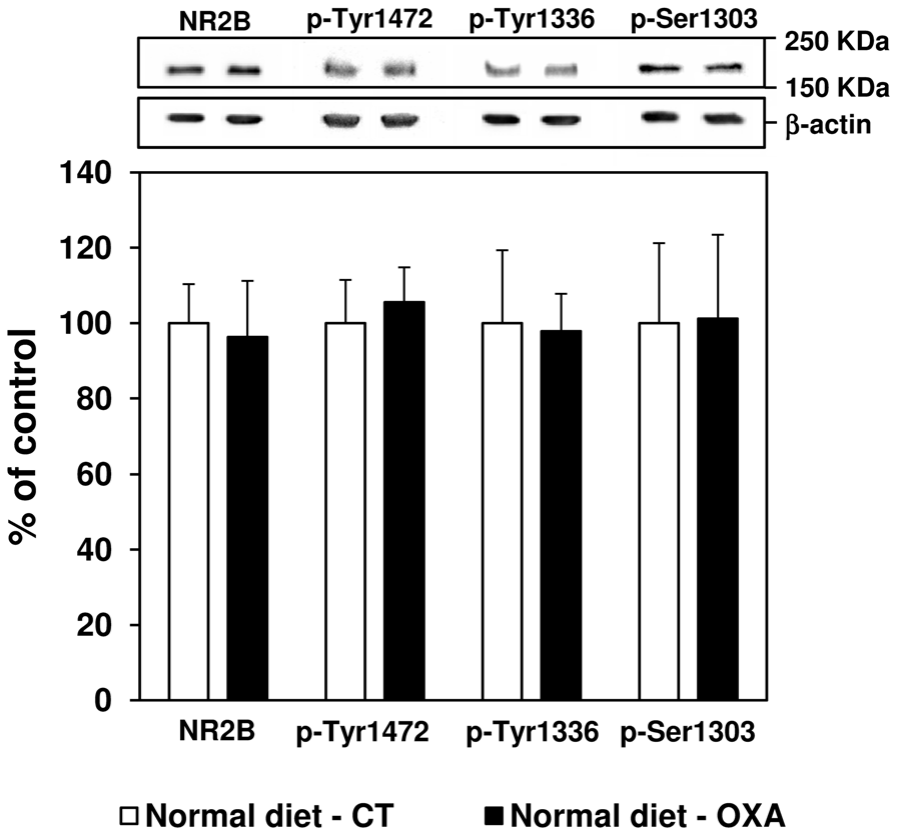
Effect of an oxaliplatin injection on spinal NR2B protein expression and phosphorylation at day 3 in rats fed with a normal diet. Variations (%) in total NR2B, p-Tyr1472, p-Tyr1336 and p-Ser1303 NR2B expression were measured in the spinal cord dorsal horn of control rats (normal diet - CT, white bars) and oxaliplatin-treated rats (6 mg/kg, i.p., normal diet - OXA, black bars) 3 days after oxaliplatin administration. Ratios on beta-actin are expressed as means ± SEM. Representative western blots are shown for NR2B/phospho-NR2B (upper panel) and the corresponding beta-actin signals (lower panel). A non-parametric Mann-Whitney-Wilcoxon test was used to assess statistical differences between groups.

At the same time after oxaliplatin injection, as shown in Figure 4, immunoblot analysis revealed no change in NR2B subunit expression compared to control rats. Furthermore, levels of p-Tyr1472, p-Tyr1336 and p-Ser1303 NR2B subunits remained unchanged after oxaliplatin injection compared to control rats, at day 3.

#### The PDD suppresses oxaliplatin-induced increase of glutamate level

The PDD, devoid of any effect by itself on glutamate level, suppressed the oxaliplatin-induced increase in Glu concentration at day 3 post-oxaliplatin injection and the decrease in tCr (Table 1). For oxaliplatin-treated rats, spinal Glu concentration was significantly lower in rats fed with a PDD compared to a normal diet (−59%, p = 0.0143), as well as concentrations of glutamate precursors: glutamine (Gln) (−16% p = 0.0143) and N-acetylaspartylglutamate (NAAG) (−24%, p = 0.0283). A significant decrease in aspartate (Asp) (−36%, p = 0.0283), another excitatory amino-acid, was also observed in the oxaliplatin-treated rats fed with a PDD compared to a normal diet, as well as a decrease of its precursor N-acetylaspartate (NAA) (−24%, p = 0.0143).

### The PDD Induces Other Metabolic Variations in the Spinal Cord

At day 3, in control rats fed with a PDD, there was a significant decrease in PtC (−25%, p = 0.0143), GPC (−22%, p = 0.0143), AXP (−22%, p = 0.0143), alanine (Ala) (−19%, p = 0.0283), lactate (Lac) (−15%, p = 0.0209) and myo-inositol (MyI) (−15%, p = 0.0275) concentrations compared to control rats fed with a normal diet. Inversely Ace levels increased (+25%, p = 0.0339) in response to PDD compared to control rats fed with a normal diet (Table 1). Similarly, PLS-DA evidenced a marked separation of control rats according to diet (R2Y = 0.646, Q2 = 0.547, validated model, Figure 3-B) and the most discriminant metabolites explaining this separation were AXP, beta-hydroxybutyrate (BHB), Lac, PtC and tCr.

At the same time, in oxaliplatin-treated rats fed with a PDD, the concentrations of succinate (Succ) (−59%, p = 0.0472) and choline-derivatives metabolites phosphocholine (PC) (−13%, p = 0.0472), PtC (−19%, p = 0.0163) and GPC (−19%, p = 0.0163) decreased significantly compared to oxaliplatin-treated rats fed with a normal diet (Table 1). PLS-DA also showed a clear separation between the diets for oxaliplatin-treated rats (R_2_Y = 0.649, Q_2_ = 0.504, validated model, Figure 3-C), indicating that the metabolic phenotype of these two groups is significantly distinct. This separation was attributed to Ala, Gln, Lac, MyI and NAA.

### Ifenprodil, a NR2B-selective Antagonist, Relieves Oxaliplatin-induced Mechanical and Cold Hypersensitivity

As a PDD reduced the oxaliplatin-induced increase in spinal Glu concentration, we suspected that a direct inhibition of the spinal glutamate/NR2B-containing NMDA receptors pathway would be effective to reduce oxaliplatin-induced hypersensitivity. Thus we assessed the effect of an intrathecal injection of NR2B-specific antagonist, ifenprodil, on oxaliplatin-induced mechanical and cold hypersensitivity.

**Figure 5:**
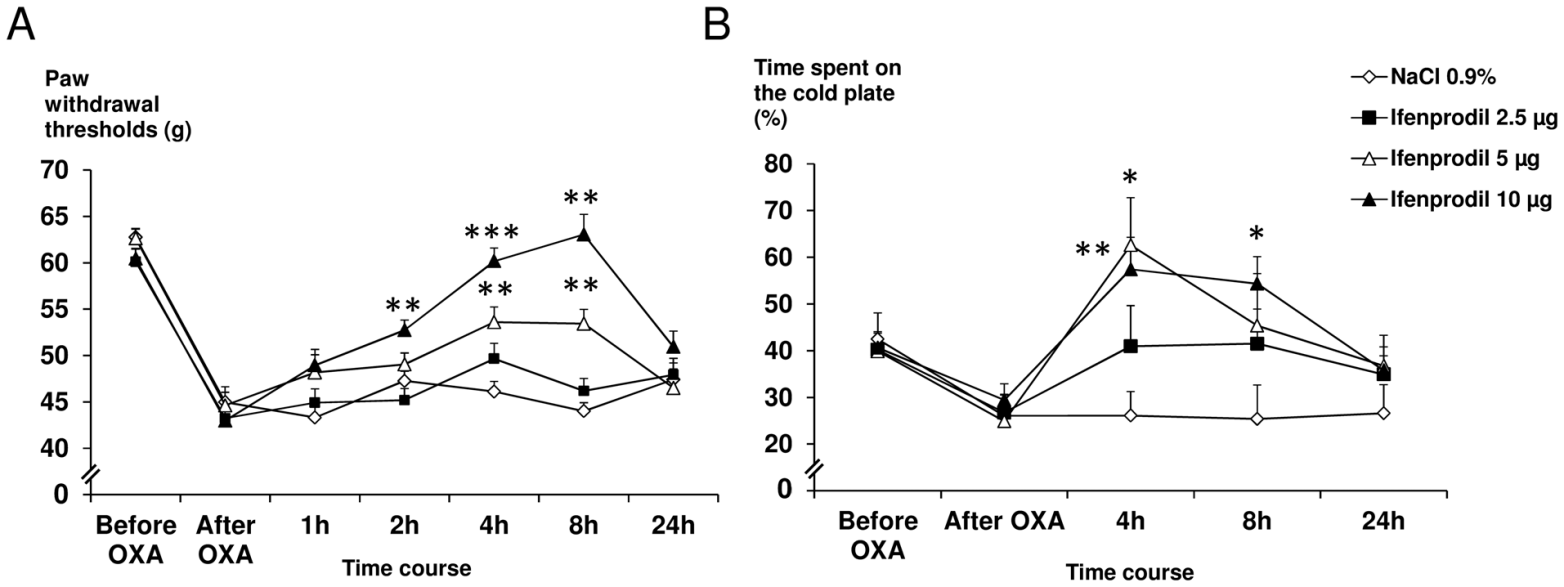
Effect of ifenprodil on oxaliplatin-induced mechanical and cold hypersensitivity at day 3 in rats fed with a normal diet. Neuropathic pain was induced in rats fed with normal diet by a single i.p. injection of oxaliplatin (OXA) at 6 mg/kg on day 0. (A) Mechanical hypersensitivity was assessed before and 3 days after oxaliplatin injection, and then at 1, 2, 8 and 24 h after an intrathecal injection of ifenprodil (2.5, 5 and 10 µg), using the electronic von Frey test. (B) Cold hypersensitivity expressed as time spent on the cold plate (%) was assessed before and 3 days after oxaliplatin injection and then at 4, 8 and 24 h after an intrathecal injection of ifenprodil (2.5, 5 and 10 µg) using the thermal place preference test (12°C *vs*. 25°C). All data are expressed as means ± SEM (n = 6 to 9 per group). Two-way repeated-measures ANOVA followed by a Tukey-Kramer *post hoc* test. *, p<0.05; **, p<0.01; ***, p<0.001 *vs*. NaCl 0.9%.

In the electronic von Frey test, while 2.5 µg ifenprodil/rat was inactive, 5 µg/rat induced a significant increase in paw withdrawal thresholds from 4 h to 8 h compared to controls (+16%, p = 0.006 and +21%, p = 0.003, respectively). Rats treated with 10 µg ifenprodil/rat showed a significant increase in paw withdrawal thresholds at 2 h (+12%, p = 0.005), 4 h (+31%, p<0.001) and 8 h (+43%, p = 0.004) compared to saline-treated rats and oxaliplatin-induced mechanical hypersensitivity was totally reversed, at 4 h and 8 h (mechanical thresholds were not significantly different from pre-oxaliplatin values, p = 0.90 and p = 0.24, respectively). At 24 h post-ifenprodil administration, paw withdrawal thresholds were not significantly different from the saline group regardless of the dose (Figure 5-A).

Similarly, in the thermal place preference test, the first active dose of ifenprodil was 5 µg/rat with a significant increase (+140%, p = 0.02) in the time spent on the cold plate, at 4 h, compared to control rats. Rats treated with 10 µg ifenprodil/rat showed a significant increase in the time spent on the cold plate at 4 h (+120%, p = 0.001) and 8 h (+114%, p = 0.01) compared to control rats. Moreover, the time spent on the cold plate was significantly higher than pre-oxaliplatin values in rats treated with 10 µg/rat at 4 h (+35%, p = 0.017) and 8 h (+28%, p = 0.045). At 24 h post-ifenprodil administration, time spent on the cold plate was not significantly different from the saline control group regardless of the dose (Figure 5-B).

## Discussion

Up to 96% of patients experience acute cold hypersensitivity after oxaliplatin administration [20]. Even if oxaliplatin-based chemotherapy remains the reference treatment for advanced colorectal cancers [1], its neurotoxicity is often a cause for early chemotherapy discontinuation and greatly impairs patient quality of life [25].

This study reports that a 7-day PDD was able to prevent oxaliplatin-induced acute cold and mechanical hypersensitivity in rats and brings novel findings on the molecular mechanisms enabling PDD to prevent oxaliplatin-induced neurotoxicity.

The antinociceptive effect of the PDD might result from a decrease in spinal NMDA receptor activity as demonstrated by Rivat *et al.* [13]. Increased spinal NR2B subunit expression and/or phosphorylation has been associated with the development of pain behavior in a wide range of animal pain models, including inflammation [26], traumatic neuropathy [27], toxic neuropathy [28] and cancer pain [29]. However, in the present study, there was no variation in NR2B protein expression or tyrosine and serine phosphorylation in response to oxaliplatin injection. These results are in accordance with Mihara *et al.* (2011), who did not find any variation in NR2B expression (gene and protein) in the rat spinal dorsal horn 5 days after oxaliplatin injections (4 mg/kg i.p., day 1 and 2). An increase of NR2B expression was only found following repeated administration of oxaliplatin over 25 days (4 mg/kg i.p. twice a week, cumulative dose: 32 mg/kg) [8]. On the other hand, even though we failed to show an increase in NR2B activity, we demonstrated a hyperactivity of the glutamate pathway in the spinal dorsal horn following an acute injection of oxaliplatin. This hyperactivity, illustrated by the increased concentration of glutamate in the spinal dorsal horn, was reversed by the PDD simultaneously with the induced hyperalgesia, which suggests a causal link between the two changes. Indeed, glutamate is long known to be a major neurotransmitter of sensory input between primary afferent fibers and nociceptive ascending pathways [30]. Furthermore, numerous data from literature establish a link between increase in spinal glutamate levels and pain hypersensitivity in chronic pain models (e.g. an increase of spinal glutamate was shown in a rat model of trauma-induced painful neuropathy [31]. Moreover, antagonists of NMDA receptors are effective in patients with neuropathic pain [12], [32].

The metabolic analysis performed here allows the evaluation of the biochemical impact of PDD on glutamate biosynthesis. Glutamate is synthetized in neurons from 2-oxoglutarate originating from the tricarboxylic acid cycle [33]. In oxaliplatin-treated rats fed with a PDD, the spinal level of succinate (a tricarboxylic acid cycle intermediate) is significantly lower than in oxaliplatin-treated rats fed with a normal diet, suggesting that the absence of increased glutamate concentration in oxaliplatin-treated rats fed with a PDD could result from a decreased activity of the tricarboxylic acid cycle in spinal neurons. This hypothesis is supported by the significant decrease in adenosine phosphate concentration in control rats fed with a PDD. Interestingly, significant decreases in glutamine and NAAG (respectively the astrocyte [34] and neuron [35] storage form of glutamate) were also observed, suggesting that the PDD may interact with several metabolic pathways to prevent oxaliplatin-induced increase in spinal glutamate biosynthesis. Another potential precursor of glutamate is ornithine (through the concerted action of ornithine aminotransferase and glutamate-5-semialdehyde dehydrogenase or the 1-pyrroline-5-carboxylate dehydrogenase [33]), which is also a precursor of polyamines [15]. It is possible that the polyamine deficiency induces an increased *de novo* synthesis of polyamines (through activation of ODC which is dependent upon polyamine uptake [36]), thus depleting ornithine from neurons and preventing glutamate synthesis from this metabolite. The decreased lactate concentration observed in rats fed with a PDD (control and oxaliplatin-treated) could indicate an alteration of aerobic glycolysis in astrocytes, which is directly dependent on glutamate uptake [37]. Finally, we observed a significant decrease of glycerophosphocholine and phosphatidylcholine in rats fed with a PDD (regardless of the treatment received) compared to rats fed with a normal diet (regardless of the treatment received). Neurodegenerative diseases are commonly associated with an increase of these metabolites in the nervous system [38]. Moreover, polyamines have been shown to interact with phospholipids and to stabilize membrane formation [39]. We could suspect that the PDD might play a role of neuroprotection which, in combination with the decrease in glutamate levels, could participate to the prevention of acute pain hypersensitivity induced by oxaliplatin.

Intrathecal injection of ifenprodil induced an antihyperalgesic effect in conditions of oxaliplatin-induced mechanical and cold hypersensitivity. This result, with recent previous ones obtained with ifenprodil (i.e. an antihyperalgesia in a rat model of chronic dorsal root ganglia compression [27] or in oxaliplatin-induced mechanical allodynia [8] confirms the involvement of an increased, NR2B-dependent, activity of the spinal glutamate system in the pathophysiology of oxaliplatin-induced neuropathic pain symptoms (cold and mechanical hypersensitivity). Together with the metabolic and antihyperalgesic effects of the PDD, these results show that oxaliplatin-induced hypersensitivity can be reduced by inhibiting the glutamate pathway either upstream through a decrease in glutamate level (PDD effect) or downstream by an antagonism of the glutamate target (ifenprodil action).

Whilst a PDD is able to prevent the acute oxaliplatin induced cold and mechanical hypersensitivity, we could also put forward the hypothesis that a PDD could also prevent oxaliplatin-induced chronic neuropathy, associated with repeated oxaliplatin administrations. Considering that the thermal hyperalgesia is a predictive marker of the chronic neuropathy [20], this PDD could ultimately provide neuroprotection against oxaliplatin-induced chronic neuropathy. However, the hypothetic preventive effect of a PDD on the oxaliplatin-induced chronic neuropathy must be assessed in the same experimental conditions in an animal model reproducing the symptomatology of this chronic neuropathy such as described by Ling et al. [40]. In this chronic neuropathy, NR2B antagonists (ifenprodil and Ro25-6981) decreased mechanical allodynia [8]. Moreover, excessive release of glutamate (which could be induced by repeated cycles of oxaliplatin) in CNS is known to be responsible for the excitotoxicity associated with neuronal damage (e.g. in spinal inhibitory neurons) and death [41]. The pharmacological inhibition of the glutamate carboxypeptidase, which hydrolyses N-acetylaspartylglutamate (NAAG) into N-acetylaspartate (NAA) and glutamate, decreased glutamate level and improved nerve conduction velocity as well as morphological alterations in dorsal root ganglia of animal models of cisplatin- and bortezomib-induced neuropathy [42].

To our knowledge, this is the first study to report an increased glutamate concentration in the spinal dorsal horn of rats after one injection of oxaliplatin and to report that a PDD prevented both this increase and the mechanical and thermal hypersensitivity. Managing oxaliplatin-induced neurotoxicity is of crucial importance in oncology, as this toxicity is often a cause of dose reduction or treatment discontinuation in cancer patients. Thus, reducing polyamine dietary intake for these patients could represent a safe and effective strategy to prevent the acute sensory disturbances associated with oxaliplatin infusions. A clinical trial (NCT01775449) is currently recruiting patients to assess this new hypothesis.

## References

[1] de Gramont A, Figer A, Seymour M, Homerin M, Hmissi A, et al (2000) Leucovorin and fluorouracil with or without oxaliplatin as first-line treatment in advanced colorectal cancer. J Clin Oncol 18: 2938–2947. 10944126 10.1200/JCO.2000.18.16.2938

[2] Gamelin E, Gamelin L, Bossi L, Quasthoff S (2002) Clinical aspects and molecular basis of oxaliplatin neurotoxicity: current management and development of preventive measures. Semin Oncol 29: 21–33. 10.1053/sonc.2002.35525 12422305

[3] Pasetto LM, D’Andrea MR, Rossi E, Monfardini S (2006) Oxaliplatin-related neurotoxicity: how and why? Crit Rev Oncol Hematol 59: 159–168. 16806962 10.1016/j.critrevonc.2006.01.001

[4] Park SB, Lin CS, Krishnan AV, Goldstein D, Friedlander ML, et al (2011) Long-term neuropathy after oxaliplatin treatment: challenging the dictum of reversibility. Oncologist 16: 708–716. 21478275 10.1634/theoncologist.2010-0248 PMC3228192

[5] Tofthagen C (2010) Surviving chemotherapy for colon cancer and living with the consequences. J Palliat Med 13: 1389–1391. 21091028 10.1089/jpm.2010.0124

[6] Tofthagen C, Overcash J, Kip K (2012) Falls in persons with chemotherapy-induced peripheral neuropathy. Support Care Cancer 20: 583–589. 21380613 10.1007/s00520-011-1127-7 PMC5386000

[7] Gent P, Massey K (2001) An overview of chemotherapy-induced peripheral sensory neuropathy, focusing on oxaliplatin. Int J Palliat Nurs 7: 354–359. 11951404 10.12968/ijpn.2001.7.7.9020

[8] Mihara Y, Egashira N, Sada H, Kawashiri T, Ushio S, et al (2011) Involvement of spinal NR2B-containing NMDA receptors in oxaliplatin-induced mechanical allodynia in rats. Mol Pain 7: 8. 21247499 10.1186/1744-8069-7-8 PMC3033350

[9] Boyce S, Wyatt A, Webb JK, O’Donnell R, Mason G, et al (1999) Selective NMDA NR2B antagonists induce antinociception without motor dysfunction: correlation with restricted localisation of NR2B subunit in dorsal horn. Neuropharmacology 38: 611–623. 10340299 10.1016/s0028-3908(98)00218-4

[10] De Vry J, Kuhl E, Franken-Kunkel P, Eckel G (2004) Pharmacological characterization of the chronic constriction injury model of neuropathic pain. Eur J Pharmacol 491: 137–148. 15140630 10.1016/j.ejphar.2004.03.051

[11] Qu XX, Cai J, Li MJ, Chi YN, Liao FF, et al (2009) Role of the spinal cord NR2B-containing NMDA receptors in the development of neuropathic pain. Exp Neurol 215: 298–307. 19046970 10.1016/j.expneurol.2008.10.018

[12] Parsons CG (2001) NMDA receptors as targets for drug action in neuropathic pain. Eur J Pharmacol 429: 71–78. 11698028 10.1016/s0014-2999(01)01307-3

[13] Rivat C, Richebe P, Laboureyras E, Laulin JP, Havouis R, et al (2008) Polyamine deficient diet to relieve pain hypersensitivity. Pain 137: 125–137. 17900809 10.1016/j.pain.2007.08.021

[14] Milovic V (2001) Polyamines in the gut lumen: bioavailability and biodistribution. Eur J Gastroenterol Hepatol 13: 1021–1025. 11564949 10.1097/00042737-200109000-00004

[15] Moinard C, Cynober L, de Bandt JP (2005) Polyamines: metabolism and implications in human diseases. Clin Nutr 24: 184–197. 15784477 10.1016/j.clnu.2004.11.001

[16] Williams K (1997) Interactions of polyamines with ion channels. Biochem J 325 (Pt 2): 289–297. 10.1042/bj3250289 PMC1218558 9230104

[17] Kergozien S, Bansard JY, Delcros JG, Havouis R, Moulinoux JP (1996) Polyamine deprivation provokes an antalgic effect. Life Sci 58: 2209–2215. 8649207 10.1016/0024-3205(96)00215-9

[18] Gaboriau F, Havouis R, Moulinoux JP, Delcros JG (2003) Atmospheric pressure chemical ionization-mass spectrometry method to improve the determination of dansylated polyamines. Anal Biochem 318: 212–220. 12814624 10.1016/s0003-2697(03)00214-8

[19] Ling B, Coudore-Civiale MA, Balayssac D, Eschalier A, Coudore F, et al (2007) Behavioral and immunohistological assessment of painful neuropathy induced by a single oxaliplatin injection in the rat. Toxicology 234: 176–184. 17418472 10.1016/j.tox.2007.02.013

[20] Attal N, Bouhassira D, Gautron M, Vaillant JN, Mitry E, et al (2009) Thermal hyperalgesia as a marker of oxaliplatin neurotoxicity: a prospective quantified sensory assessment study. Pain 144: 245–252. 19457614 10.1016/j.pain.2009.03.024

[21] Binder A, Stengel M, Maag R, Wasner G, Schoch R, et al (2007) Pain in oxaliplatin-induced neuropathy–sensitisation in the peripheral and central nociceptive system. Eur J Cancer 43: 2658–2663. 17855072 10.1016/j.ejca.2007.07.030

[22] Daulhac L, Mallet C, Courteix C, Etienne M, Duroux E, et al (2006) Diabetes-induced mechanical hyperalgesia involves spinal mitogen-activated protein kinase activation in neurons and microglia via N-methyl-D-aspartate-dependent mechanisms. Mol Pharmacol 70: 1246–1254. 16868181 10.1124/mol.106.025478

[23] Angenstein F, Hilfert L, Zuschratter W, Altrock WD, Niessen HG, et al (2008) Morphological and metabolic changes in the cortex of mice lacking the functional presynaptic active zone protein bassoon: a combined 1H-NMR spectroscopy and histochemical study. Cereb Cortex 18: 890–897. 17652465 10.1093/cercor/bhm122

[24] Verbeke G, Molenberghs G (2009) Linear Mixed Models for Longitudinal Data. New York: Springer Verlag.

[25] Balayssac D, Ferrier J, Descoeur J, Ling B, Pezet D, et al (2011) Chemotherapy-induced peripheral neuropathies: from clinical relevance to preclinical evidence. Expert Opin Drug Saf 10: 407–417. 21210753 10.1517/14740338.2011.543417

[26] Guo W, Zou S, Guan Y, Ikeda T, Tal M, et al (2002) Tyrosine phosphorylation of the NR2B subunit of the NMDA receptor in the spinal cord during the development and maintenance of inflammatory hyperalgesia. J Neurosci 22: 6208–6217. 12122079 10.1523/JNEUROSCI.22-14-06208.2002 PMC6757905

[27] Zhang W, Shi CX, Gu XP, Ma ZL, Zhu W (2009) Ifenprodil induced antinociception and decreased the expression of NR2B subunits in the dorsal horn after chronic dorsal root ganglia compression in rats. Anesth Analg 108: 1015–1020. 19224818 10.1213/ane.0b013e318193ffd2

[28] Narita M, Miyoshi K, Suzuki T (2007) Changes in function of NMDA receptor NR2B subunit in spinal cord of rats with neuropathy following chronic ethanol consumption. Life Sci 80: 852–859. 17156796 10.1016/j.lfs.2006.11.015

[29] Gu X, Zhang J, Ma Z, Wang J, Zhou X, et al (2011) The role of N-methyl-D-aspartate receptor subunit NR2B in spinal cord in cancer pain. Eur J Pain 14: 496–502. 10.1016/j.ejpain.2009.09.001 19815434

[30] Millan MJ (1999) The induction of pain: an integrative review. Prog Neurobiol 57: 1–164. 9987804 10.1016/s0301-0082(98)00048-3

[31] Kawamata M, Omote K (1996) Involvement of increased excitatory amino acids and intracellular Ca2+ concentration in the spinal dorsal horn in an animal model of neuropathic pain. Pain 68: 85–96. 9252003 10.1016/S0304-3959(96)03222-8

[32] Fisher K, Coderre TJ, Hagen NA (2000) Targeting the N-methyl-D-aspartate receptor for chronic pain management. Preclinical animal studies, recent clinical experience and future research directions. J Pain Symptom Manage 20: 358–373. 11068158 10.1016/s0885-3924(00)00213-x

[33] Torgner I, Kvamme E (1990) Synthesis of transmitter glutamate and the glial-neuron interrelationship. Mol Chem Neuropathol 12: 11–17. 1980584 10.1007/BF03160053

[34] Hertz L, Dringen R, Schousboe A, Robinson SR (1999) Astrocytes: glutamate producers for neurons. J Neurosci Res 57: 417–428. 10440891

[35] Fuhrman S, Palkovits M, Cassidy M, Neale JH (1994) The regional distribution of N-acetylaspartylglutamate (NAAG) and peptidase activity against NAAG in the rat nervous system. J Neurochem 62: 275–281. 8263527 10.1046/j.1471-4159.1994.62010275.x

[36] Seiler N, Dezeure F (1990) Polyamine transport in mammalian cells. Int J Biochem 22: 211–218. 2110083 10.1016/0020-711x(90)90332-w

[37] Pellerin L, Magistretti PJ (1994) Glutamate uptake into astrocytes stimulates aerobic glycolysis: a mechanism coupling neuronal activity to glucose utilization. Proc Natl Acad Sci U S A 91: 10625–10629. 7938003 10.1073/pnas.91.22.10625 PMC45074

[38] Klein J (2000) Membrane breakdown in acute and chronic neurodegeneration: focus on choline-containing phospholipids. J Neural Transm 107: 1027–1063. 11041281 10.1007/s007020070051

[39] Bachrach U (2005) Naturally occurring polyamines: interaction with macromolecules. Curr Protein Pept Sci 6: 559–566. 16381604 10.2174/138920305774933240

[40] Ling B, Authier N, Balayssac D, Eschalier A, Coudoré F (2007) Behavioral and pharmacological description of oxaliplatin-induced painful neuropathy in rat. Pain 128: 225–234. 17084975 10.1016/j.pain.2006.09.016

[41] Rzeski W, Pruskil S, Macke A, Felderhoff-Mueser U, Reiher AK, et al (2004) Anticancer agents are potent neurotoxins in vitro and in vivo. Ann Neurol 56: 351–360. 15349862 10.1002/ana.20185

[42] Carozzi VA, Chiorazzi A, Canta A, Lapidus RG, Slusher BS, et al (2010) Glutamate carboxypeptidase inhibition reduces the severity of chemotherapy-induced peripheral neurotoxicity in rat. Neurotox Res 17: 380–391. 19763734 10.1007/s12640-009-9114-1

